# Revealing spatiotemporal inequalities, hotspots, and determinants in healthcare resource distribution: insights from hospital beds panel data in 2308 Chinese counties

**DOI:** 10.1186/s12889-024-17950-y

**Published:** 2024-02-09

**Authors:** Chao Song, Lina Fang, Mingyu Xie, Zhangying Tang, Yumeng Zhang, Fan Tian, Xiuli Wang, Xiaojun Lin, Qiaolan Liu, Shixi Xu, Jay Pan

**Affiliations:** 1 HEOA Group, West China School of Public Health and West China Fourth Hospital, Sichuan University, Chengdu, Sichuan China; 2 Institute for Healthy Cities and West China Research Centre for Rural Health Development, Sichuan University, Chengdu, Sichuan China; 3 West China-PUMC C.C. Chen Institute of Health, Sichuan University, Chengdu, Sichuan China; 4 School of Public Health, Xi’an Jiaotong University, Xi’an, Shaanxi China; 5 State Key Laboratory of Oil and Gas Reservoir Geology and Exploitation, School of Geoscience and Technology, Southwest Petroleum University, Chengdu, Sichuan China; 6 China Center for South Asian Studies, Sichuan University, Chengdu, Sichuan China

**Keywords:** Healthcare resources, Spatiotemporal heterogeneity, Small area, Inequality, Hotspots, Determinants, Hospital beds, China

## Abstract

**Background:**

Ensuring universal health coverage and equitable access to health services requires a comprehensive understanding of spatiotemporal heterogeneity in healthcare resources, especially in small areas. The absence of a structured spatiotemporal evaluation framework in existing studies inspired us to propose a conceptual framework encompassing three perspectives: spatiotemporal inequalities, hotspots, and determinants.

**Methods:**

To demonstrate our three-perspective conceptual framework, we employed three state-of-the-art methods and analyzed 10 years’ worth of Chinese county-level hospital bed data. First, we depicted spatial inequalities of hospital beds within provinces and their temporal inequalities through the spatial Gini coefficient. Next, we identified different types of spatiotemporal hotspots and coldspots at the county level using the emerging hot spot analysis (Getis-Ord Gi* statistics). Finally, we explored the spatiotemporally heterogeneous impacts of socioeconomic and environmental factors on hospital beds using the Bayesian spatiotemporally varying coefficients (STVC) model and quantified factors’ spatiotemporal explainable percentages with the spatiotemporal variance partitioning index (STVPI).

**Results:**

Spatial inequalities map revealed significant disparities in hospital beds, with gradual improvements observed in 21 provinces over time. Seven types of hot and cold spots among 24.78% counties highlighted the persistent presence of the regional Matthew effect in both high- and low-level hospital bed counties. Socioeconomic factors contributed 36.85% (95% credible intervals [CIs]: 31.84–42.50%) of county-level hospital beds, while environmental factors accounted for 59.12% (53.80–63.83%). Factors’ space-scale variation explained 75.71% (68.94–81.55%), whereas time-scale variation contributed 20.25% (14.14–27.36%). Additionally, six factors (GDP, first industrial output, local general budget revenue, road, river, and slope) were identified as the spatiotemporal determinants, collectively explaining over 84% of the variations.

**Conclusions:**

Three-perspective framework enables global policymakers and stakeholders to identify health services disparities at the micro-level, pinpoint regions needing targeted interventions, and create differentiated strategies aligned with their unique spatiotemporal determinants, significantly aiding in achieving sustainable healthcare development.

**Supplementary Information:**

The online version contains supplementary material available at 10.1186/s12889-024-17950-y.

## Introduction

Enhancing the equitable distribution of healthcare resources is crucial for achieving universal health coverage and the Sustainable Development Goals (SDGs), with a particular focus on SDG 3 [1–3]. Small-area evaluation, which pertains to evaluating at a relatively smaller geographic scale (e.g., counties within a country, census tracts within a city, etc.), yields statistically more robust results of under- or unobserved geographic units and reveals local heterogeneity [4]. In small areas, geospatial inequalities in healthcare resources are most pronounced [5, 6] and vary more significantly over time, underscoring the necessity of considering small-area spatiotemporal heterogeneity in both evaluations and interventions [7, 8]. However, current mainstream evaluations, primarily grounded in spatial perspectives, have failed to sufficiently assess healthcare resource inequalities in small areas within spatiotemporal dimensions, thus hindering targeted policy interventions that cater to local time-specific conditions [9, 10]. It is imperative to evaluate small-area healthcare resource distribution from a spatiotemporal heterogeneity perspective. Unfortunately, there is a lack of a common conceptual evaluation framework as a guide to support effective and context-specific allocation of healthcare resources across both spatial and temporal scales [11, 12].

The geospatial evaluation for healthcare resources encompasses three primary dimensions: identifying spatial inequalities, detecting geographic hotspots, and exploring local influencing factors [13]. Although a variety of methods have been developed to support these geospatial evaluations, few evaluation methods consider spatiotemporal heterogeneity. To begin with, spatial inequalities evaluation pertains to assessing the level of equilibrium in healthcare resource distribution across diverse geographical scales [14]. Traditional studies have utilized non-spatial methods, such as the Gini coefficient [15], to demonstrate persistent inequalities in healthcare resource allocation [16, 17]. However, these non-spatial methods disregard the impact of spatial autocorrelation, which contravenes the first law of geography, leading to uncertainties in their findings [18, 19]. To surmount this, researchers have proposed the spatial Gini coefficient to identify spatial inequalities in healthcare resources [20]. For instance, Kalogious employed it to assess spatial inequalities in healthcare institution distribution in Greece, revealing compelling evidence of the spatial autocorrelation effect and inequalities in healthcare institution distribution [21]. Xu et.al examined the spatial inequality of prenatal diagnostic services in China, discovering higher levels of spatial inequality in the central and eastern regions than in the western regions [22]. Regrettably, studies on healthcare resource spatial inequalities evaluations have frequently overlooked the crucial temporal dimension, highlighting the necessity for comprehensive spatiotemporal assessments.

The evaluation of geographic hotspots extends beyond spatial inequalities by explicitly identifying specific spatial types of spatial clusters, reflecting the regional Matthew effect in healthcare resource distribution [23, 24], which suggests that wealthier regions tend to receive more healthcare resources, leaving poorer regions facing scarcity [25]. While researchers have employed tools such as local Moran’s *I* index to assess geographic hotspots, these methods are invalid in capturing their dynamic temporal characteristics [26–28]. Currently, the space-time scan statistics have gained wide application in public health due to their ability to detect spatial clusters of varying sizes and locations [29]. The space-time scan statistics can mainly detect the likelihood of clusters, differentiating between the “most likely clusters”, “secondary clusters”, and others [30, 31], however, this method cannot precisely specify the exact category of these identified clusters, such as hot or cold spots. To address this, a novel approach called emerging hot spot analysis has emerged, enabling the detection of patterns of spatiotemporal clusters (hot and cold spots) and categorizing them according to their temporal trends [32, 33], which is of utmost importance for comprehending the prevailing conditions and trends of healthcare resource allocation across different areas [34]. Unfortunately, existing research has primarily applied this method to explore spatiotemporal hotspot patterns in environmental health and infectious diseases [35, 36], with limited application in the context of health services research.

The evaluation of local factors influencing healthcare resources delves deeper into the potential reasons behind spatial inequalities and hotspots, laying the foundation for identifying key determinants for future interventions [37, 38]. Prior research has highlighted the substantial impact of socioeconomic factors, such as GDP and residents’ saving deposits, on the spatial distribution of healthcare resources [39, 40]. Furthermore, environmental factors, including the normalized vegetation index, are also acknowledged as influential drivers shaping the spatial allocation of healthcare resources [41]. While these analyses primarily relied on global regression models like ordinary least squares regression and spatial econometric models, it is important to note that such global regressions may not adequately account for spatially heterogeneous associations between variables, known as spatial non-stationarity. This can lead to errors in specific small areas [42]. Consequently, researchers have turned to local spatial regression models, such as geographically weighted regression, to identify spatial heterogeneity in the factors influencing healthcare resources in small areas [43, 44]. These models have revealed that slope, local general budget revenue, and topography have varying impacts on healthcare resources across different districts [45, 46].

More importantly, given the dynamic variation of local healthcare resources, it is essential to explore the spatiotemporal non-stationarity of influencing factors. Nevertheless, methods addressing spatiotemporal non-stationarity remain relatively rare and complex [47]. Among these methods, the Bayesian spatiotemporally varying coefficients (STVC) model serves as a unified full-map approach for detecting spatiotemporal non-stationarity [48]. It has been employed to confirm significant spatiotemporally heterogeneous associations between hospital bed resources and socioeconomic (e.g., urban worker population density, total investment in fixed assets) and environmental conditions (e.g., wind speed, river density) within two regions of China [49, 50]. Beyond the identification of spatiotemporally heterogeneous influence effects of potential factors, the more important concern lies in pinpointing the key determinants for healthcare resources, as they provide the foundation for potential intervention strategies. The spatiotemporal variance partitioning index (STVPI) offers an advanced means of evaluating the relative percentage contribution of each explanatory factor after accounting for spatiotemporal non-stationarity, aiming to elucidate pivotal spatiotemporal determinants [51]. Unfortunately, no studies to date have discovered the key spatiotemporal determinants driving small-area healthcare resources by assessing the contributions of influencing factors over space and time.

Given the scarcity of comprehensive assessments that simultaneously integrate these three dimensions (inequalities, hotspots, and determinants), particularly from a spatiotemporal perspective, we are inspired to propose a novel conceptual framework for healthcare resource assessment, grounded in the spatiotemporal heterogeneity observed among small areas. This framework facilitates precise measurement of equity and offers flexible policy guidance tailored to local conditions, addressing the pronounced healthcare resource disparities within small areas. More importantly, we incorporate state-of-the-art methods for each dimension into our framework, including the spatial Gini coefficient, the emerging hot spot analysis, and the Bayesian STVC model along with its STVPI, to evaluate spatiotemporal inequalities, hotspots, and determinants of small-area healthcare resource distribution, respectively.

In China, despite substantial investments in healthcare resources by the Chinese government, the national-level feat of improvement belied the persistent disparities at the sub-national level [52–54]. However, extant studies have primarily focused on disparities at the provincial level [41, 55, 56], overlooking small-area spatiotemporal inequalities [57]. Given that counties serve as the cornerstone of healthcare system reform in China and represent the smallest administrative unit for healthcare resource allocation [58, 59], our representative case centers on county-level hospital beds in China. Hospital beds also stand out as a pivotal indicator among healthcare resources, symbolizing the capacity of healthcare services and mirroring healthcare institutions’ capability to provide inpatient care and treatment [60, 61]. Therefore, we utilized 10-year hospital beds panel data from 2308 counties in China to illustrate the effectiveness of our framework. This work also marks the first application of our spatiotemporal conceptual framework to county-level healthcare resources in China, aiming to promote a more rational distribution in small areas and contribute to the realization of universal health coverage and the achievement of SDGs.

## Materials and methods

### Data sources

This study collected a 10-year panel dataset from 2308 Chinese counties, covering the period from 2002 to 2011. The primary variable of interest is the small-area hospital beds. In Fig. S1, we illustrate the distribution of county-level hospital beds, using data from the year 2011 as an example. The health resource density index (HRDI) is selected as the response variable of hospital beds to avoid bias caused by a single demographic or geographic factor. HRDI is the geometric mean of the number of hospital beds per 1000 population and square kilometers [62]. Its formula is as follows: $HRDI=\sqrt{\left({y}_i/{P}_i\right)\left({y}_i/{A}_i\right)}$, where *y*_*i*_ represents the health resource of the unit *i*, *P*_*i*_ represents the population of the unit *i*, and *A*_*i*_ represents the area of the unit *i*.

Based on the previous literature review [38, 49, 63], we incorporated a total of 32 socioeconomic and environmental variables as potential influencing factors, as outlined in Table S1. The data on hospital beds and socioeconomic factors were retrieved from a published dataset of China’s county-level official socioeconomic statistics, originally collected from the China County Statistical Yearbook, the China Statistical Yearbook for Regional Economy, and the China City Statistical Yearbook [64]. The environmental data are sourced from the National Meteorological Information Center (http://data.cma.cn/) and the Resources and Environment Science and Data Center (http://www.resdc.cn/). Nevertheless, due to the discontinuation of certain county-level socioeconomic indicators in China post-2013 [64], we were constrained to utilize data spanning only from 2002 to 2011 to preserve key socioeconomic determinants that may drive county-level healthcare resources.

Variance inflation factor (VIF) is employed to detect multicollinearity among factors [65]. To identify variables with lower multicollinearity, we set a VIF threshold of 5. The overall contribution of influencing factors can be determined using the increase in node purity indicator calculated by Random Forest: the higher the value, the greater the relative contribution of potential variables to hospital bed distribution [66]. It is worth noting that, unlike Bayesian STVC modeling, the Random Forest method does not consider the spatiotemporal heterogeneity in the impact of factors, which could potentially introduce bias and uncertainty [51].

### Spatiotemporal conceptual framework for assessing healthcare resources

**Fig. 1:**
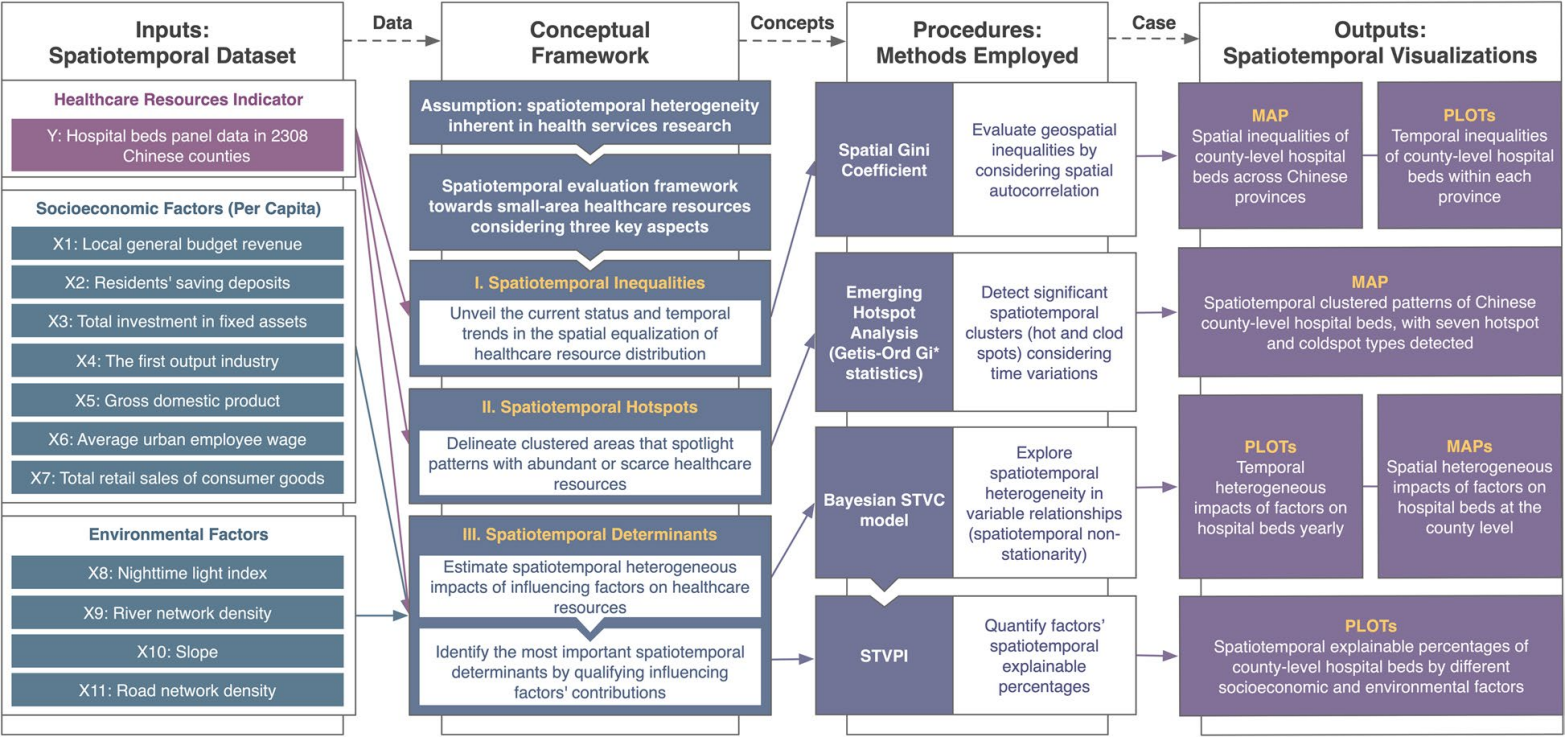
Flowchart for assessing spatiotemporal inequalities, hotspots, and determinants in healthcare resource distribution: using Chinese county-level hospital beds as an example. STVC: spatiotemporally varying coefficients. STVPI: spatiotemporal variance partitioning index

Our study explores the multifaceted nature of small-area healthcare resources assessment by introducing three core assessment perspectives: spatiotemporal inequalities, spatiotemporal hotspots, and spatiotemporal determinants, as illustrated in Fig. 1. Specifically, spatiotemporal inequalities assessment unveils the current status and temporal trends in the spatial equalization of healthcare resource distribution across small areas. Spatiotemporal hotspots assessment delineates small areas that spotlight patterns with abundant or scarce healthcare resources, thereby highlighting anomalous areas that require targeted interventions. Furthermore, by detecting the spatiotemporal heterogeneous impacts of influencing factors and quantifying their space-time contributions, we can uncover key determinants for supporting tailored, time-specific optimization strategies aligned with these determinants to promote equality in small-area healthcare resource distribution.

To achieve the three evaluation dimensions, we employed advanced geospatial statistical methods, focusing on China’s county-level hospital beds as a case study. To be specific, we employed the spatial Gini coefficient to dynamically assess annual spatiotemporal inequalities in hospital bed distribution. We applied the emerging hot spot analysis to identify geographic clustered patterns (hot and cold spots) that take into account time-changing features to pinpoint counties demanding particular attention. Additionally, we adopted the Bayesian STVC model and STVPI to unravel the spatiotemporal heterogeneous impacts of socioeconomic and environmental factors on county-level hospital bed distribution and determine their spatiotemporal relative contribution percentages. The following sections elaborate on the statistical mechanics and implementation of these methods within our spatiotemporal assessment framework.

#### Spatial Gini coefficient

The spatial Gini coefficient, an advanced statistic that considers spatial autocorrelation effects, is a robust measure for evaluating spatial inequalities in healthcare resources, generating more accurate and reliable results [20]. It relies on the spatial weight matrix to determine whether two areas are neighbors and calculates the neighbor, non-neighbor, and spatial Gini coefficients. Here, “neighbors” refers to counties that share a geographical border on at least one side. The global spatial Gini coefficient is obtained by adding the neighbor and non-neighbor Gini coefficients. To calculate the spatial Gini coefficient, one can use the lctools package in R [21]. Equation (1) elucidates the calculation of the spatial Gini coefficient.

1 $$G=\frac{\sum_{i=1}^n{\sum}_{j=1}^n{w}_{i,j}\left|{y}_i-{y}_j\right|}{2{n}^2\overline{y}}+\frac{\sum_{i=1}^n{\sum}_{j=1}^n\left(1-{w}_{i,j}\right)\left|{y}_i-{y}_j\right|}{2{n}^2\overline{y}}$$

where *y*_*i*_ and *y*_*j*_ are the values of hospital beds in counties *i* and *j*, respectively. *n* is the number of counties within each province. $\overline{y}=\frac{1}{n}\sum \limits_{i=1}^n{y}_i$ represents the mean value of *y*_*i*_. *w*_*i*, *j*_ are the weights that are usually 1 for neighbor observations and 0 for non-neighbor observations. The spatial Gini coefficient falls within the same range (0–1) as the Gini coefficient. Higher values of the spatial Gini coefficient indicate greater disparities between counties, reflecting more pronounced inter-regional inequalities.

#### Emerging hot spot analysis

The emerging hot spot analysis, achieved through the integration of spatial and temporal data into a cube, identifies locations characterized by significantly higher or lower values of a variable compared to the overall distribution [36]. It categorizes areas into various types of spatial clustered patterns, determined by the presence of hot and cold spots and their temporal trends. We utilized the Emerging Hot Spot Analysis tool in ArcGIS Pro software to evaluate spatiotemporal hotspot patterns. The emerging hot spot analysis is based on the Getis-Ord Gi* statistics, which is calculated using eqs. (2) and (3):

2 $${G_i}^{\ast }=\frac{\sum \limits_{j=1}^n{w}_{i,j}{y}_j-\overline{y}\sum \limits_{j=1}^n{w}_{i,j}}{s\sqrt{\frac{\left[n{\sum}_{j=1}^n{w}^2_{~i,j}-{\left(\sum \limits_{j=1}^n{w}_{i,j}\right)}^2\right]}{n-1}}}$$

3 $$\overline{y}=\frac{\sum_{j=1}^n{w}_j}{n},s=\sqrt{\frac{\sum_{j=1}^n{y}_i^2}{n}-{\left(\overline{y}\right)}^2}$$

where *w*_*i*, *j*_ represents the spatial weight among counties *i* and *j*. *y*_*j*_ is the value of hospital beds *y* in county *j*. *n* is the total number of counties. *s* is the standard deviation of hospital beds. A hot spot emerges when the degree of aggregation of healthcare resources in the research area increases with the absolute value of the *G*_*i*_^∗^ index. The *G*_*i*_^∗^ index is positive for the high-value clustering areas (hot spots) and negative for the low-value clustering areas (cold spots).

#### Bayesian STVC model

The Bayesian spatiotemporally varying coefficients (STVC) model represents a unified full-map approach to detecting the inherent spatiotemporal heterogeneity in variable relationships, which is also known as spatiotemporal non-stationarity [48]. The model estimates a series of local parameters that vary over time and space, enabling the identification of spatiotemporal heterogeneous impacts of different independent variables on the dependent variable, while accounting for spatiotemporal autocorrelations. Equations (4) and (5) illustrate how a common type of STVC model works for this case.

4 $$\log \left({y}_{it}\right)=\sum \limits_{k=1}^K{\mu}_{ik}{X}_{it k}+\sum \limits_{k=1}^K{\gamma}_{tk}{X}_{it k}+{\varepsilon}_{it}$$

5 $${\mu}_{ik}\left|{\mu}_{- ik}\sim N\left({\overline{\mu}}_{w_i,k},\frac{\sigma_{\mu k}^2}{n_{w_i,k}}\right),{\gamma}_{tk}\right|{\gamma}_{t-1,k},{\gamma}_{t-2,k}\sim N\left(2{\gamma}_{t-1,k}+{\gamma}_{t-2,k},{\sigma}_{\gamma k}^2\right),{\varepsilon}_{it}\sim N\left(0,{\sigma}_{\varepsilon}^2\right)$$

Here, *y*_*it*_ is the indicator of hospital beds *y* in the county *i* and year *t*. The response *y*_*it*_ is assumed to follow the log-Gaussian distribution from an exponential family, and its conditional mean is linked to the additive linear predictor through a natural logarithmic function [67]. Independent variables *X*, having variations in spatial and temporal dimensions, represent *K* socioeconomic and environmental factors that influence *y*. Local parameters *μ*_*ik*_ are referred to as space-coefficients (SCs) for the *k*-th *X*, while local parameters *γ*_*tk*_ are denoted as the *k*-th *X* ‘s time-coefficients (TCs). *ε*_*it*_ represent the modelling residuals.

All items incorporated in this particular STVC model are treated as random effects. Two types of prior latent Gaussian models (LGM) are used to estimate the posterior spatial and temporal random effects. As shown in Eq. (5), the *X* ‘s spatial non-stationary random effect *μ*_*ik*_ is assigned a popular prior spatial LGM called the Conditional Autoregressive (CAR) model, where ${\overline{\mu}}_{w_i}={n}_{w_i}^{-1}{\sum}_{j\in {w}_i}{\mu}_j$, *w*_*i*_ and ${n}_{w_i}$ denote the set of spatial neighbors (where spatial adjacencies are considered neighbors) and the number of neighbors of the county *i*. Correspondingly, the *X* ‘s temporal non-stationary random effect *γ*_*tk*_ is assigned a widely-used prior temporal LGM known as the Random Walk (RW) model, with a second-order type applied in this case. *ε*_*it*_ is assumed to be independent and identically distributed.

#### Spatiotemporal variance partitioning index

The Bayesian STVC modeling introduces the spatiotemporal variance partitioning index (STVPI) to further assess the proportion of explainable variation in space-time attributed to each factor [51]. Equation (6) demonstrates the calculation of the STVPI, quantifying the percentage of variation of the target variable explained by each spatiotemporally heterogeneous influencing factor.

6 $${\rho}_k=\frac{\sigma_{\mu k}+{\sigma}_{\gamma k}}{\sum_{k=1}^K\left({\sigma}_{\mu k}+{\sigma}_{\gamma k}\right)+{\sigma}_{\varepsilon }}\times 100\%$$

where *ρ*_*k*_ is expressed as a percentage in the range of [0,100] for the *k*-th explanatory factor. *σ*_*μk*_ represents the variance component of the spatially non-stationary random effect of the *k*-th explanatory factor at the standard deviation scale. The *k*-th explanatory factor’s temporally non-stationary random effect is represented by *σ*_*γk*_ at the standard deviation scale. *σ*_*ε*_ denotes the variance component of the unexplained random effect in the residual term.

The STVPI offers flexibility by allowing adjustments to the combination of random effects in the numerator of Eq. (6). This flexibility enables the inclusion of additional evaluation dimensions and leads to a more nuanced understanding of the spatiotemporal impacts of factors [48]. Here, the STVPI can determine the contribution percentage of influencing factors in separate time, separate space, and spatiotemporal coupling dimensions. It also specifies the percentage of spatiotemporal variability in hospital beds explained by the Bayesian STVC model and residual, socioeconomic, and environmental factors, as well as space and time scales.

## Results

### Spatiotemporal inequalities of Chinese hospital beds

We implemented the spatial Gini coefficient to measure the spatial inequalities in the distribution of hospital beds across Chinese counties from 2002 to 2011, where a lower value suggested a more equal distribution of hospital beds spatially. We calculated three types of spatial Gini coefficients, including global, neighbor, and non-neighbor Gini coefficients, alongside their respective proportions (%) (Table S2). The neighbor Gini coefficients accounted for a range of 0.16 to 7.65% of the global Gini coefficients, indicating the existence of spatial autocorrelation among county-level hospital beds. The spatial Gini coefficient exhibited higher in non-neighbor districts, indicating that the distribution of county-level hospital beds was also influenced by non-neighbor areas.

**Fig. 2:**
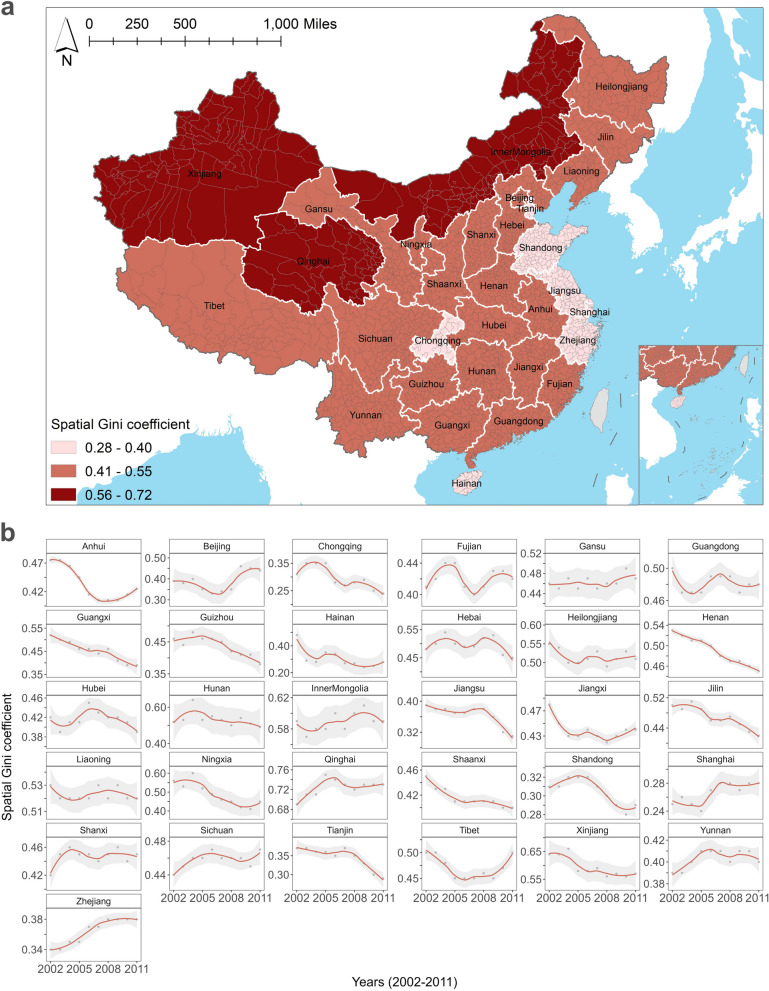
Spatial Gini coefficient: (**a**) accessing spatial inequalities and (**b**) temporal inequalities in county-level hospital bed distribution across Chinese provinces

In the spatial dimension, we produced an average spatial Gini coefficients map (Fig. 2a) to visualize the 10-year average spatial inequalities of hospital beds across Chinese provinces. Red represents the highest inequalities, orange indicates moderate inequalities, and pink represents a relatively equal distribution. Notably, six provinces namely Chongqing, Tianjin, Shandong, Jiangsu, Zhejiang, and Hainan, exhibited a relatively equal spatial distribution of hospital beds, while Xinjiang Uyghur Autonomous Region, Inner Mongolia Autonomous Region, and Qinghai Province showed higher levels of inequalities. In the temporal dimension (Fig. 2b), 21 provinces (67.74%) experienced a significant decrease in the spatial Gini coefficients, suggesting an improvement in the equalities of the spatial distribution of hospital beds. Among these provinces, Chongqing, Jiangsu, Shandong, and Tianjin consistently maintained a balanced distribution. In addition, 10 provinces (32.26%) displayed an upward trend in inequalities. The trend in Gansu showed a relatively gradual increase, while Beijing and Inner Mongolia demonstrated a pattern of initially declining trends followed by a subsequent increase. Conversely, the trend exhibited an initial rise followed by a subsequent decline in Fujian. To sum up, despite the overall decrease in county-level hospital bed inequalities within most provinces over 10 years, our spatiotemporal fine-scale evaluation revealed persistent disparities in the spatial dimension.

### Spatiotemporal hotspots of Chinese hospital beds

**Fig. 3:**
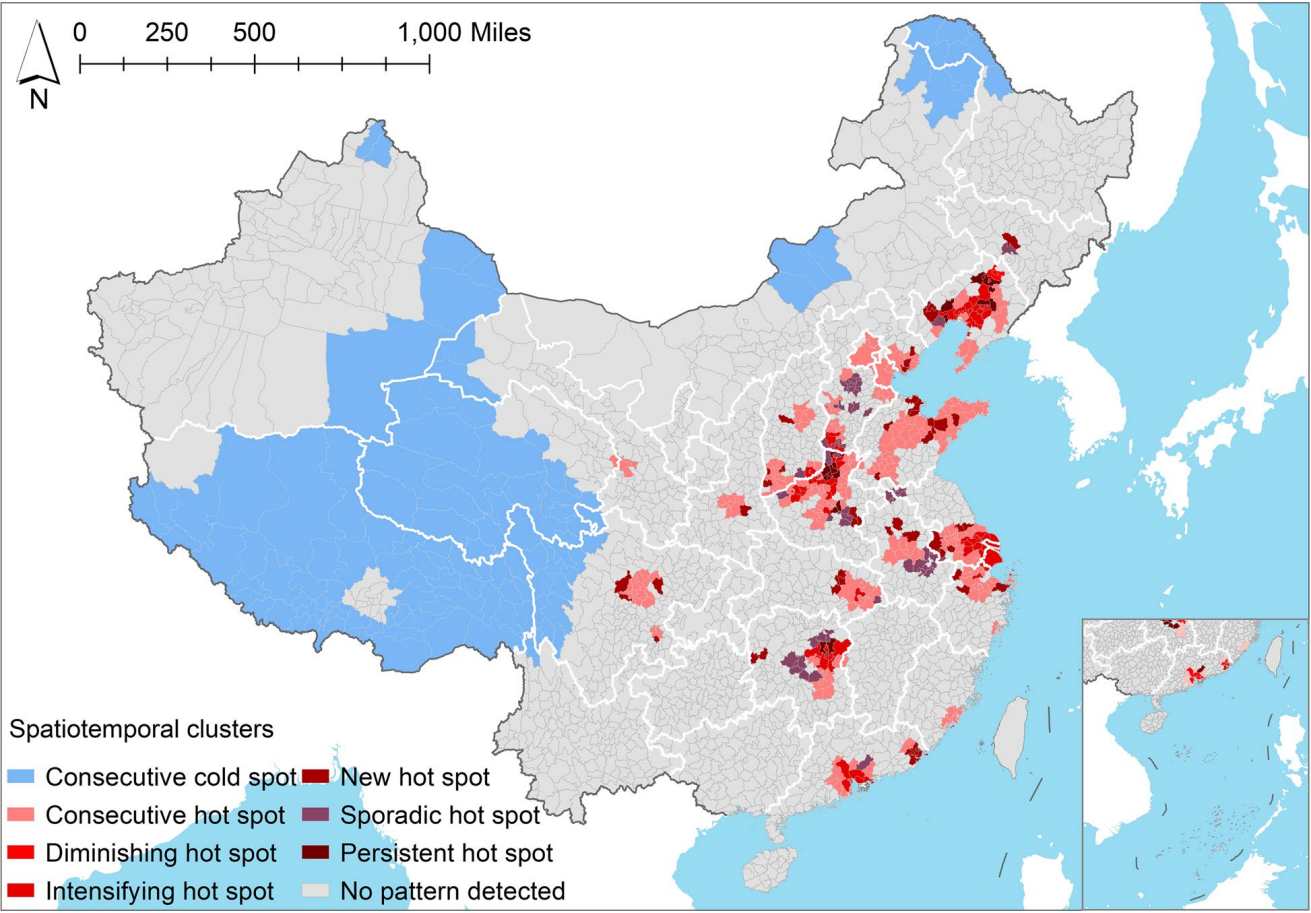
Spatiotemporal hotspot patterns of Chinese county-level hospital beds detected with the emerging hot spot analysis. Consecutive cold spot: Hospital beds consistently at a low level. Consecutive hot spot: Hospital beds consistently at a high level. Diminishing hot spot: Initially high hospital bed levels with a statistically significant decrease in intensity over an extended period. Intensifying hot spot: Hospital beds consistently at a high level for at least 9 years with a statistically significant increase in intensity. New hot spot: Hospital beds showing high values for the first time in the last year, previously not identified as hot spots. Sporadic hot spot: Statistically significant hotspots of hospital beds occurring intermittently and irregularly over multiple years. Persistent hot spot: Sustained high intensity of hospital beds over a long period without a statistically significant increase

Through the use of emerging hotspot analysis, we further identified multiple spatial clustered patterns (hot and cold spots) of hospital beds, taking into account their temporal trends (Fig. 3). The explanations of these spatiotemporal hotspot patterns were summarized in Table S3. Hot spots indicated counties that signified elevated hospital beds, reaching statistical significance, while cold spots denoted counties characterized by a decline in hospital beds that attained statistical significance. The terms “consecutive, diminishing, intensifying, persistent, sporadic, and new hot spots” further signify spatiotemporal trends in the prevalence of these spots’ patterns. Among all counties, 572 counties (24.78%) exhibited significant spatiotemporal clusters that could be categorized into seven types of hot or cold spot patterns. The number of hotspot counties (452, 79.02%) exceeded that of coldspot counties (120, 20.98%), suggesting that there was still a large inequality in counties despite the overall trend of growth in hospital bed resources.

Delving further into these spatiotemporal variations, we uncovered six types of hot spot patterns, namely consecutive, diminishing, intensifying, persistent, sporadic, and new hot spots. Conversely, only the consecutive cold spot pattern was identified. We further elaborated on the intricacies of the spatiotemporal distribution characteristics for each pattern. To commence, the dominant pattern was the consecutive hot spot pattern, signifying a sustained high level of county-level hospital beds over an extended period, observed in 257 counties (44.93%), primarily located in East, North, and Central areas. Secondly, the diminishing hot spot patterns (2, 0.35%) were observed only in Kaiyuan and Tieling counties, indicating that the hospital beds remained high level for a long time, but eventually decreased. The intensifying hot spot patterns (65, 11.36%) predominantly manifested in the central and eastern parts. The pattern indicated a significant spatial hotspot occurred in at least nine out of 10 studied years, with a statistically significant increase over time. Furthermore, the new hot spot patterns (high values had never been observed before) in 50 counties (8.74%), were primarily concentrated in the central and eastern regions. The persistent hot spot patterns (maintained high values for an extended period without any statistically significant increase) were identified in 20 counties (3.50%), scattered across Liaoning, Henan, and Hunan provinces. Lastly, 58 counties (10.14%) with sporadic hot spot patterns were mainly concentrated in Hebei and Henan, indicating the remarkable spatial hotspot occurred intermittently across different years. The bipolar distribution of hospital bed hotspots across China highlighted an insufficiency in the improvement of county-level healthcare resource spatial distribution over the decade, necessitating further attention and remedial action on scarcity areas.

### Spatiotemporal determinants of Chinese hospital beds

A total of 32 socioeconomic and environmental variables were collected as potential influencing factors for China’s county-level hospital beds. Initially, we used the VIF to identify and remove factors with a VIF exceeding 5, as shown in Fig. S2a. Subsequently, we ranked the remaining factors based on the increase in node purity indicator using Random Forest, selecting those with a higher increase in node purity. Finally, we retained 11 factors that demonstrated a substantial contribution to county-level hospital beds. For clarity, we renumbered the 11 influencing factors as X1 to X11 (Fig. S2b). These factors include local general budget revenue per capita (X1), residents’ saving deposits per capita (X2), total investment in fixed assets per capita (X3), first industry output per capita (X4), GDP per capita (X5), average urban employee wage (X6), total retail sales of consumer goods per capita (X7), nighttime light index (X8), river network density (X9), slope (X10), and road network density (X11). We utilized these factors to construct the Bayesian STVC model and obtain their spatiotemporal contributions with STVPI.

#### Temporal heterogeneous associations between factors and hospital beds

**Fig. 4:**
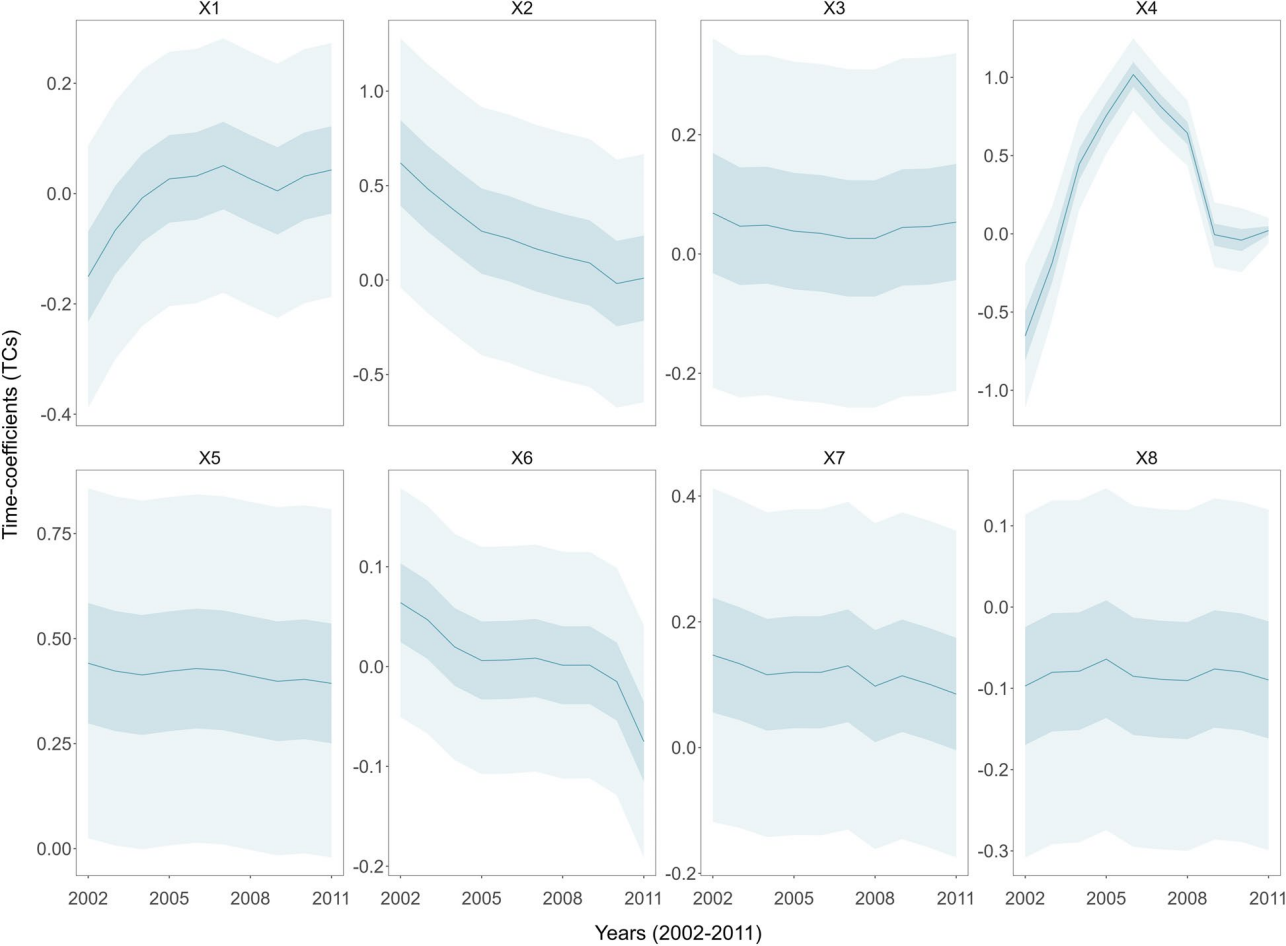
Temporally heterogeneous associations of hospital beds with influencing factors (X1-X8) during 2002–2011, using time-coefficients (TCs) estimated via the Bayesian STVC model. Influencing factors encompass a range of variables, including local general budget revenue per capita (X1), residents’ saving deposits per capita (X2), total investment in fixed assets per capita (X3), first industry output per capita (X4), GDP per capita (X5), average urban employee wage (X6), total retail sales of consumer goods per capita (X7), and nighttime light index (X8). Median (50%), narrow (25–75%), and wide (2.5–97.5%) credible intervals (CIs) are represented using varying levels of transparency

With the Bayesian STVC model, we examined the temporally varying impacts of eight socioeconomic and environmental factors on the provision of hospital beds across Chinese counties, as shown in Fig. 4, using time-coefficients (TCs) parameters along with wide and narrow credible intervals (CIs) for each factor. Remarkably, all factors exhibited significant time-varying trends in their impacts, highlighting the temporal non-stationarity of their impacts on county-level hospital beds. Specifically, the impacts of residents’ saving deposits per capita (X2), total investment in fixed assets per capita (X3), and GDP per capita (X5) exhibited linear trends in hospital beds. Furthermore, the impacts of local general budget revenue per capita (X1) presented yearly increases; and the impacts of first output industry per capita (X4) first rose and then declined, but overall increased. The impacts of nighttime light index (X8) remained relatively stable over time. In contrast, the TCs of residents’ saving deposits per capita (X2), total investment in fixed assets per capita (X3), GDP per capita (X5), average urban employee wage (X6), and total retail sales of consumer goods per capita (X7) exhibited overall downward trends, indicating the decrement in their impacts on hospital beds as time progressed.

#### Spatial heterogeneous associations between factors and hospital beds

**Fig. 5:**
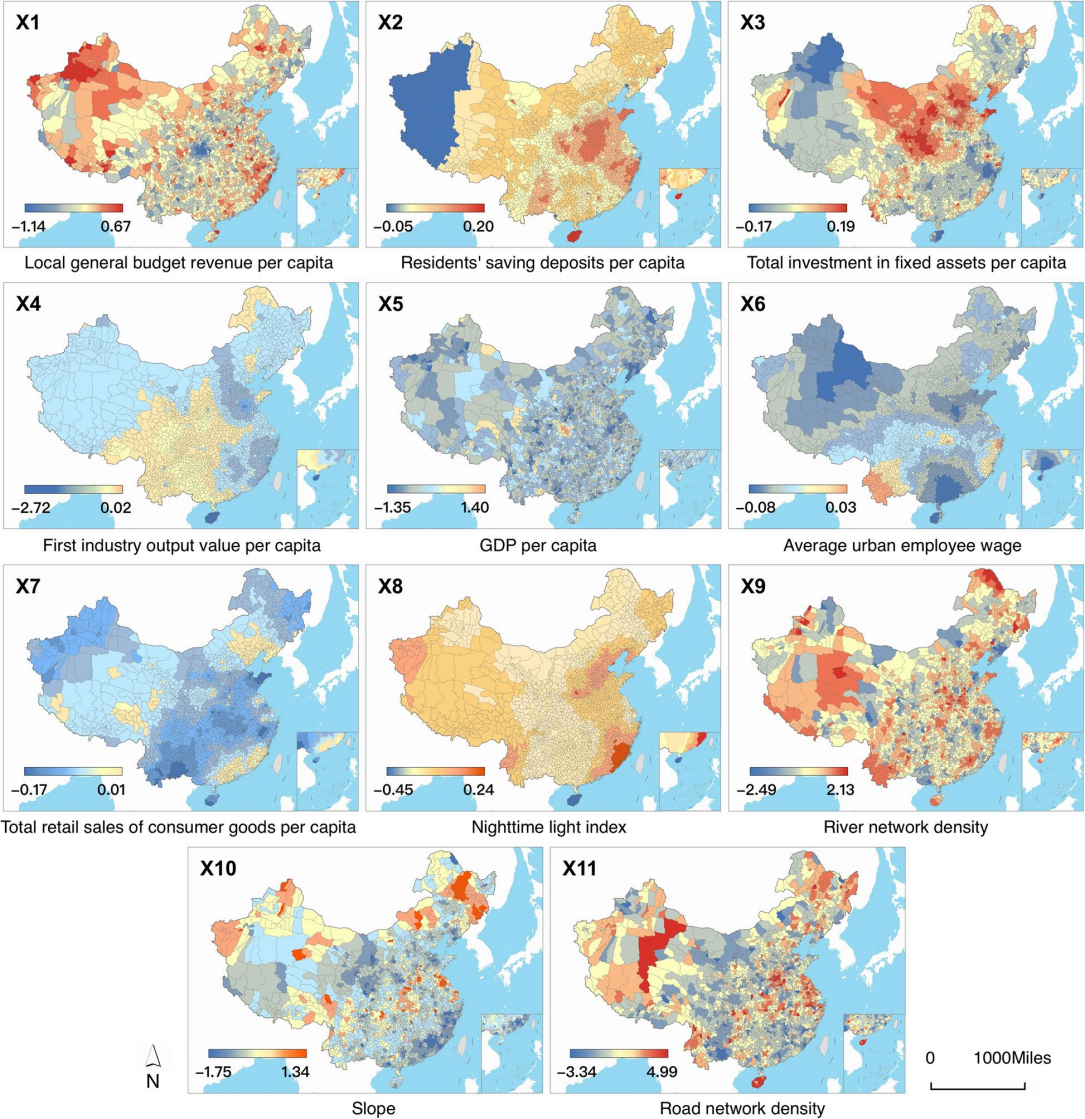
Spatially heterogeneous associations of hospital beds with influencing factors (X1-X11) during 2002–2011, mapped by Bayesian STVC-estimated space-coefficients (SCs)

To spatially represent the county-level influences of socioeconomic and environmental factors, we utilized space-coefficients (SCs) parameters from the Bayesian STVC model to capture the spatially heterogeneous associations of 11 factors with hospital beds, as depicted in Fig. 5. In the figure, red indicates positive correlations, while blue denotes negative correlations. The spatial impacts of six factors on hospital beds exhibited a strong regularity at the county level: residents’ saving deposits per capita (X2), total investment in fixed assets per capita (X3), first industry output value per capita (X4), average urban employee wage (X6), total retail sales of consumer goods per capita (X7), and nighttime light index (X8). In contrast, the spatial impacts of the other five factors displayed weaker regularity, suggesting the potential for tailored policies and measures in specific counties based on these factors. The series of spatial non-stationarity maps (SCs) demonstrated significant spatial heterogeneity in the impacts of different explanatory factors on hospital beds at the county level, underscoring the importance of adapting policies to the local conditions in China.

#### Percentage contributions of spatiotemporally heterogeneous influencing factors

**Fig. 6:**
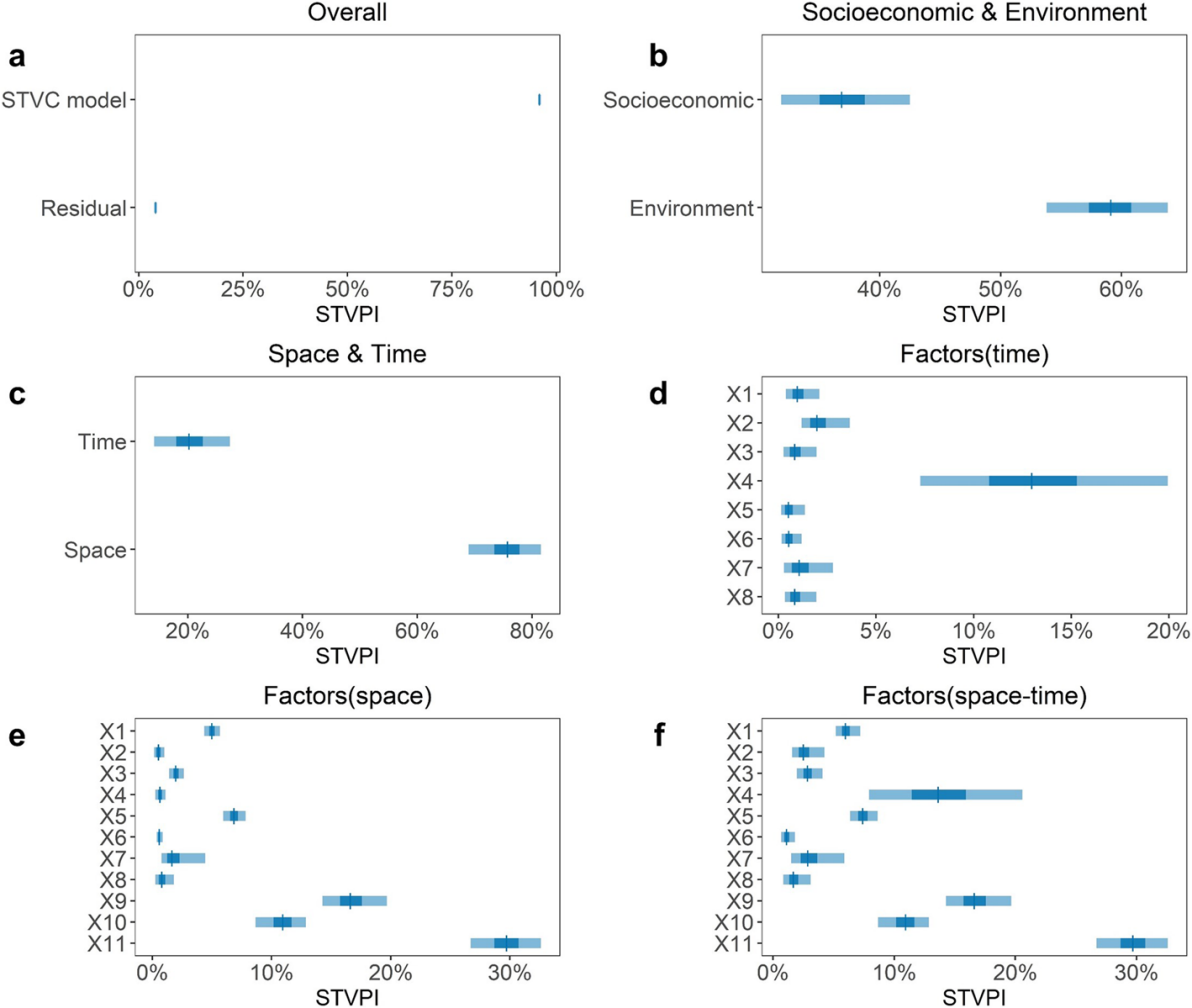
Spatiotemporal percentage contributions of explanatory factors (X1-X11) on Chinese county hospital beds, quantified by the spatiotemporal variance partitioning index (STVPI). **a** contribution percentages explained by the Bayesian STVC model and the residual; **b** contribution percentages of socioeconomic and environmental factors considering their spatiotemporal heterogeneous associations with hospital beds; **c** contribution percentages of the space and time scales concerning all factors; **d** time-scale contribution percentage for each factor; **e** space-scale contribution percentage for each factor; and **f** space-time-scale contribution percentage for each factor. Explanatory factors include local general budget revenue per capita (X1), residents’ saving deposits per capita (X2), total investment in fixed assets per capita (X3), first industry output per capita (X4), GDP per capita (X5), average urban employee wage (X6), total retail sales of consumer goods per capita (X7), nighttime light index (X8), river network density (X9), slope (X10), and road network density (X11)

Upon detecting the spatiotemporal heterogeneous impacts of socioeconomic and environmental factors, we further used the STVPI to quantify the relative importance (percentage contributions) of these explanatory factors across six dimensions (Fig. 6 and Table S4). On the whole, the results (Fig. 6a) demonstrate that the selected factors under the spatiotemporal non-stationary assumption can explain a substantial portion of the variation in Chinese county-level hospital beds, with the STVC model explaining 95.96% (95% CIs: 95.63–96.33%) of the variation and the residual contributing 4.04% (95% CIs: 3.67–4.37%). Environmental factors and socioeconomic factors account for 59.12% (95% CIs: 53.80–63.83%) and 36.85% (95% CIs: 31.84–42.50%) of the variation, respectively (Fig. 6b). Regarding the dimensions of space and time, the space dimension contributes 75.70% (95% CIs: 68.94–81.55%) to hospital beds, while the time dimension accounts for 20.25% (95% CIs: 14.14–27.36%) (Fig. 6c).

We further elucidated the explainable percentages of each explanatory factor in space and time scales, separately. In terms of the time scale (Fig. 6d), the first industry output per capita (X4) exhibited the highest explainable percentage at 12.97% (95% CIs: 7.26–19.94%), followed by residents’ saving deposits per capita (X2) at 1.97% (95% CIs: 1.18–3.66%). In contrast, the space scale yielded different results (Fig. 6e), with the road network density (X11) contributing the largest relative share of 29.71% (95% CIs: 26.70–32.59%) to hospital beds, followed by the river network density (X9) and the slope (X10) contributing 16.60% (95% CIs: 14.27–19.67%) and 10.94% (95% CIs: 8.65–12.86%), respectively.

Considering both space and time scales (Fig. 6f), six determinants emerged as key spatiotemporal drivers of Chinese county-level hospital beds: road network density (X11), river network density (X9), first industry output per capita (X4), slope (X10), GDP per capita (X5), and local general budget revenue per capita (X1). These determinants encompass three socioeconomic aspects and three environmental conditions. Their cumulative explainable percentage exceeded 84%, emphasizing the significant roles of both socioeconomic and environmental factors in shaping the spatiotemporal evolution of Chinese county-level hospital bed resources.

## Discussion

Evaluation of spatiotemporal heterogeneity in the distribution of healthcare resources at the small-area level has been a relatively understudied aspect in current research [11, 68]. In this context, we conducted a comprehensive study using 10 years of county-level hospital bed panel data from China as a case study to introduce a universal conceptual framework effectively characterizing three key dimensions of small-area healthcare resources: spatiotemporal inequalities, spatiotemporal hotspots, and spatiotemporal determinants. Our evaluation of spatiotemporal inequalities and hotspots clearly revealed significant spatiotemporal disparities in China’s county-level hospital bed resources, shedding light on issues related to geospatial equilibrium and the regional Matthew effect. Furthermore, our investigation into spatiotemporal influencing factors successfully identified six key socioeconomic and environmental determinants, laying the groundwork for subsequent directions of intervention in China. We extend our main contributions in the following aspects.

The most significant contribution of this paper lies in the introduction of a novel spatiotemporal conceptual evaluation framework for healthcare resource distribution. This innovative evaluation system offers a comprehensive approach to assessments of healthcare resources from three distinct perspectives: small-area analysis, spatiotemporal heterogeneity, and three fundamental and core evaluation dimensions (inequalities, hotspots, and determinants). This framework not only demonstrates its potential for broader application in research on healthcare resources and health services but also advances our understanding of addressing persistent inequalities at the small-area level, given the shared relevance of healthcare resources within the three core evaluation dimensions [69]. Furthermore, we have presented three state-of-the-art methods that align with the three core dimensions of this framework, enhancing its versatility for future healthcare research. Importantly, the healthcare resource assessment framework we propose is not limited to the methods employed in our study; future investigations can harness more advanced spatiotemporal integration analysis methods for a more nuanced exploration.

We further elaborate on the three evaluation dimensions encompassed by our spatiotemporal evaluation system for small-area healthcare resources. Above all, taking laws of geography into account, the geospatial assessment provides a more precise examination of spatial inequalities in healthcare resource allocation [70]. Prior studies have predominantly relied on non-spatial methodologies, failing to account for the impact of spatial autocorrelation on such resources and consequently yielding partial outcomes [71]. To address this, the spatial Gini coefficient emerges by incorporating spatial autocorrelation into calculations to capture resource distribution characteristics [20]. Based on the case of Chinese counties, we found that the overall spatial inequalities of hospital bed resources in counties improved significantly over the study period, reflecting the Chinese government’s long-standing adherence to the strategy of promoting the rational allocation of healthcare resources, especially the inception of healthcare reform in 2009 [40, 53]. Nevertheless, spatial disparities in small areas persisted and remained a challenge for health inequalities, which implied that counties may have varied responses to the same global measures of healthcare resource investments. Here, more context-specific strategies should be implemented with dynamical reevaluation to avoid the further “one-size-fits-all” problem emerging in dealing with relatively spatiotemporal inequalities [72–75], by increasing the proportion of government expenditure on health, improving the implementation of the system of graded diagnosis and treatment, and promoting the equalization of primary healthcare services.

The evaluation of spatiotemporal hotspot and coldspot patterns provides a clear picture of healthcare resource-rich and resource-poor areas over time, enabling the evaluation of the internal clusters and pinpointing particularly anomalous areas [76, 77]. Here, we applied the emerging hot spot analysis method to point out the spatiotemporal clustered patterns of hospital bed resources in Chinese counties and provide applicable suggestions for future intervention strategies. The consecutive cold spot pattern indicates the need for context-specific countermeasures, according to their specific socioeconomic and environmental factors, to prevent further widening of the healthcare resource gap, such as increasing the volume of resources in these counties. On the other hand, the new hot spot and intensifying hot spot patterns may be attributed to the spatial catch-up effect of healthcare resources in their surrounding counties, posing a challenge to the equitable allocation of county-level healthcare resources [78]. For these counties, the higher level of government needs to prioritize the provision of policy support and reduce the extreme allocation of healthcare resources. For the consecutive, sporadic, and persistent hot spot patterns, governments should dynamically reevaluate policy measures to consider local healthcare resources supply and demand to reduce the persistent spatial hotspots of healthcare resources and prevent further wastage. In short, these hot and cold spot patterns can offer specific guidance for policy measures aimed at enhancing healthcare resource distribution and narrowing county-level disparities further.

An in-depth assessment of the spatiotemporal determinants is aimed to reveal the possible key drivers behind the spatiotemporal inequalities and hotspots of healthcare resources. Spatiotemporal visualizations based on Bayesian STVC modeling suggested that both socioeconomic and environmental factors exhibited spatiotemporal heterogeneous associations with hospital bed resources at the county level across China [49, 50], reflecting the need for local policies that are adapted to the local context as well as to the current situation. Using the STVPI, we successfully quantified factors’ space-time relative contributions to identify those determinants. We confirmed that socioeconomic (36.85%) and environmental (59.12%) factors were both important for Chinese county-level hospital beds, alongside their space-scale (75.70%) and time-scale (20.25%) contributions. Our findings reveal that three socioeconomic determinants and three environmental determinants–specifically, GDP per capita, first industry output per capita, local general budget revenue per capita, road network density, slope, and river network density–have been identified as the primary drivers. Furthermore, the cumulative relative contribution of these six factors exceeds 84%.

The possible pathways through which these spatiotemporal drivers influenced healthcare resources are as follows. Both GDP per capita and local general budget revenue per capita play pivotal roles in the adjustment and optimization of the new incremental stock of healthcare resources, exerting a direct influence on the allocation of resources through financial means. A surge in GDP inherently corresponds to an amplified government commitment to augment healthcare resources, consequently fostering a marked improvement in both the quantity and quality of healthcare resources [79, 80]. The value of the first industry output in China represents the enhancement of the economic conditions of the farming populace. Consequently, it engenders a heightened demand for health services, thereby propelling further investments in healthcare resources [81, 82]. The environmental key drivers, on the other hand, represent the local traffic and infrastructure conditions. By affecting the accessibility of healthcare resources, they subsequently affect the allocation of healthcare resources [83–85]. In summary, by identifying the key determinants influencing the distribution of hospital beds at the county level in China, we encourage the government to optimize its existing local healthcare resource allocation strategies. These strategies should be tailored to the unique spatiotemporal determinants of each county, addressing spatiotemporal inequalities and hotspots, and ultimately promoting the improvement of health equality across counties.

In conclusion, when deliberating on policy implications, our spatiotemporal conceptual framework underscores the importance of integrating multifaceted spatiotemporal perspectives in the assessment of healthcare resources within smaller administrative divisions. This framework proposes a new paradigm for policy analysis and intervention, one that recognizes the spatiotemporal heterogeneity of inequalities, hotspots, and key determinants impacting the distribution of healthcare resources in small areas. It advocates for a policy-making approach tailored to the unique spatial and temporal aspects of each area’s specific inequalities and critical issues. Such a strategy is designed to enhance outcomes effectively and mitigate the intensification of spatiotemporal disparities during interventions in these regions. Building on this foundation, we propose several potential policy directions: (i) Enhanced collaboration with local communities is crucial throughout the multilevel policy development and intervention phases, ensuring that strategies are grounded in community-specific needs and insights [52]. (ii) The significance of harnessing spatiotemporal big data and cutting-edge GeoAI technologies cannot be overstated, as these tools are instrumental in precisely predicting and comprehending health needs and resource distribution in smaller areas [86]. (iii) Finally, our framework stresses the imperative for flexible policies and interventions capable of adapting to ongoing challenges brought about by climate change, environmental degradation, and social inequality, all of which substantially affect the local demand for and allocation of health resources [87]. By spatiotemporally understanding healthcare requirements and disparities, policymakers can dynamically allocate resources, design preemptive healthcare strategies, and ensure equitable healthcare access across all regions, ultimately contributing to a more resilient and responsive healthcare system globally.

Despite its contributions, our study is not without limitations. First, due to the unavailability of comprehensive socioeconomic indicators in subsequent editions of the China Statistical Yearbook [64], the panel data from China that we utilized is restricted to the period of 2002–2011. Consequently, the applicability of the identified small-area inequalities and determinants in guiding policy measures may not be entirely suitable for the current circumstances in China. Fortunately, we still observed significant spatiotemporal heterogeneity in county-level hospital bed inequalities and their determinants, thereby affirming the validity of our conceptual framework. Second, due to healthcare resource data limitations at the Chinese county level, our study exclusively focused on hospital beds, offering insights only into the hardware aspects of healthcare resources. Future research may consider incorporating multiple indicators, including human resources [88], as well as considering their qualities [89], for a more comprehensive assessment of spatiotemporal inequalities within this framework. Third, for a more accurate assessment of spatiotemporal inequalities in healthcare resources, future research should enhance the evaluation method by integrating temporal effects into the spatial Gini coefficient, thus yielding a spatiotemporal coupled Gini coefficient. Fourth, while identifying the relative percentage contribution of spatiotemporally heterogeneous influences offers multidimensional evaluation insights for our understanding of the county-level hospital beds, achieving a comprehensive sense of geospatial attribution remains a challenging task [48]. Furthermore, the absence of sensitivity analyses in our study suggests an avenue for future research. This could involve applying the conceptual framework with alternative spatiotemporal heterogeneous evaluation methods, such as the geographically and temporally weighted regression [47], within the same study area and time period to assess the robustness and generalizability of our framework. Lastly, but importantly, our conceptual framework is designed for adaptability, allowing for ongoing inclusion and assessment of the effects of healthcare resource allocation on health outcomes [90, 91].

## Conclusions

Our study proposes a three-perspective conceptual framework for evaluating small-area healthcare resources, addressing the spatiotemporal heterogeneity through three key dimensions: spatiotemporal inequalities, hotspots, and determinants. Applying this conceptual framework to a decade-long study of county-level hospital beds in China, we have demonstrated its effectiveness and offered critical insights for improving healthcare resource equality at the county level within China. Despite overall advancements in healthcare resources, our findings indicate persistent county-level spatiotemporal disparities, underscoring the need to tackle inequalities on a localized scale. The identification of seven distinct spatiotemporal hotspot and coldspot patterns in China highlights regions with either a high concentration or a dearth of resources, thereby aiding in the formulation of responsive, site-specific strategies to meet diverse healthcare requirements. Additionally, our analysis of the varying impacts of socioeconomic and environmental factors on hospital bed distribution, and the quantification of these factors’ contribution percentages in space-time, has provided a foundation for potential targeted interventions and geospatial attribution. Moreover, the versatility of our framework extends its applicability beyond hospital bed evaluation, making it a valuable tool for assessing various vital health service indicators globally.

In light of these findings, a future direction for research and policy could involve integrating advanced spatiotemporal statistical modeling and GeoAI-driven analytics into our framework [92]. This integration would enable a more dynamic and predictive understanding of how local healthcare resource needs will evolve over time, considering factors such as climate change, environmental degradation, social inequality, and emerging health challenges. Such forward-looking approaches are essential for adapting to the rapidly changing healthcare landscape and for supporting sustainable, equitable healthcare development worldwide.

### Supplementary Information

**Additional file 1: Table S1.** Indicator system for socioeconomic and environmental variables affecting county-level hospital beds. **Table S2.** Spatial Gini coefficients of hospital beds in China. **Table S3.** Output explanation: the emerging spatiotemporal hot spot analysis for hospital beds. **Table S4.** Relative importance of space-time scale explanatory factors on hospital beds from various dimensions. **Fig. S1.** Distribution of Chinese county-level hospital beds in 2011. **Fig. S2.** Selection of potential influencing factors.

## Data Availability

Please contact the corresponding authors for data requests.

## Abbreviations

VIF: Variance inflation factor
CIs: Credible intervals
SDGs: Sustainable development goals
STVC: Spatiotemporally varying coefficients
STVPI: Spatiotemporal variance partitioning index
HRDI: Health resource density index
SCs: Space-coefficients
TCs: Time-coefficients
LGM: Latent gaussian model
CAR: Conditional autoregressive
RW: Random walk
