# Supplementary material for: Revealing spatiotemporal inequalities, hotspots, and determinants in healthcare resource distribution: insights from hospital beds panel data in 2308 Chinese counties
Source: BMC Public Health. 2024 Feb 9;24:423. doi: 10.1186/s12889-024-17950-y (PMC11218403; doi:10.1186/s12889-024-17950-y)
Supplement: Supplementary file 1 — Additional file 1: Table S1. Indicator system for socioeconomic and environmental variables affecting county-level hospital beds. Table S2. Spatial Gini coefficients of hospital beds in China. Table S3. Output explanation: the emerging spatiotemporal hot spot analysis for hospital beds. Table S4. Relative importance of space-time scale explanatory factors on hospital beds from various dimensions. Fig. S1. Distribution of Chinese county-level hospital beds in 2011. Fig. S2. Selection of potential influencing factors. [file 12889_2024_17950_MOESM1_ESM.docx]

**Supplementary Appendix**

**Table S1**. Indicator system for socioeconomic and environmental variables affecting county-level hospital beds.

**Table S2**. Spatial Gini coefficients of hospital beds in China.

**Table S3**. Output explanation: the emerging spatiotemporal hot spot analysis for county-level hospital beds.

**Table S4**. Relative importance of space-time scale explanatory factors on hospital beds from various dimensions.

**Figure S1**. Distribution of Chinese county-level hospital beds in 2011.

**Figure S2**. Selection of potential influencing factors.

**Table S1**. Indicator system for socioeconomic and environmental variables affecting county-level hospital beds (SE1 to SE20 for socioeconomic variables and EX1 to EX12 for environmental variables)

| Abbreviation | Variables | Units | Data sources |
| --- | --- | --- | --- |
| SE1 | Population density | Person/km^2^ | China County Statistical Yearbook;  China Statistical Yearbook for Regional Economy;  China City Statistical Yearbook |
| SE2 | Employee population density | Person/km^2^ |  |
| SE3 | Local telephone users’ density | Person/km^2^ |  |
| SE4 | Local government budgetary expenditures per capita | Yuan |  |
| SE5 | Local general budget revenue per capita | Yuan |  |
| SE6 | Residents’ saving deposits per capita | Yuan |  |
| SE7 | The loan balance of financial institutions per capita | Yuan |  |
| SE8 | Above-scale total industrial density | Number/km^2^ |  |
| SE9 | Above-scale total industrial output value per capita | Yuan |  |
| SE10 | Total investment in fixed assets per capita | Yuan |  |
| SE11 | Junior high school student density | Person/km^2^ |  |
| SE12 | Primary school student density | Person/km^2^ |  |
| SE13 | Gross domestic product (GDP) | Million |  |
| SE14 | First industry output per capita | Yuan |  |
| SE15 | Second industry output per capita | Yuan |  |
| SE16 | Tertiary industry output per capita | Yuan |  |
| SE17 | GDP per capita | Yuan |  |
| SE18 | Urban worker population density | Person/km^2^ |  |
| SE19 | Average urban employee wage | Yuan |  |
| SE20 | Total retail sales of consumer goods per capita | Yuan |  |
| EX1 | Normalized vegetation index (NDVI) | / | National Meteorological Information Center;  Resources and Environment Science and Data Center |
| EX2 | Nighttime light index | / |  |
| EX3 | Precipitation | 0.1 mm |  |
| EX4 | Temperature | 0.1 centigrade |  |
| EX5 | Air pressure | 1 N/m^2^ |  |
| EX6 | Wind speed | m/s |  |
| EX7 | Vapour pressure | hPa |  |
| EX8 | Sunshine hours | hours |  |
| EX9 | River network density | km/km^2^ |  |
| EX10 | Elevation | Meter |  |
| EX11 | Slope | ◦ |  |
| EX12 | Road network density | km/km^2^ |  |

**Table S2**. Spatial Gini coefficients of hospital beds in China

| Province | Gini | Gini of neighbor | Gini of non-neighbor | % Gini of neighbor | % Gini of non-neighbor |
| --- | --- | --- | --- | --- | --- |
| Hebei | 0.5106 | 0.0009 | 0.5097 | 0.18% | 99.82% |
| Shanxi | 0.4519 | 0.0008 | 0.4511 | 0.19% | 99.81% |
| Liaoning | 0.5247 | 0.0010 | 0.5237 | 0.20% | 99.80% |
| Jilin | 0.4722 | 0.0016 | 0.4707 | 0.33% | 99.67% |
| Heilongjiang | 0.5195 | 0.0012 | 0.5183 | 0.23% | 99.77% |
| Shanghai | 0.2892 | 0.0221 | 0.2671 | 7.65% | 92.35% |
| Jiangsu | 0.3678 | 0.0013 | 0.3665 | 0.36% | 99.64% |
| Chongqing | 0.3268 | 0.0067 | 0.3201 | 2.05% | 97.95% |
| Henan | 0.4982 | 0.0008 | 0.4974 | 0.16% | 99.84% |
| Zhejiang | 0.3698 | 0.0011 | 0.3687 | 0.30% | 99.70% |
| Anhui | 0.4479 | 0.0012 | 0.4467 | 0.27% | 99.73% |
| Jiangxi | 0.4337 | 0.0014 | 0.4323 | 0.31% | 99.69% |
| Inner Mongolia | 0.5931 | 0.0012 | 0.5919 | 0.20% | 99.80% |
| Hunan | 0.5463 | 0.0014 | 0.5449 | 0.25% | 99.75% |
| Guangdong | 0.4883 | 0.0013 | 0.4870 | 0.26% | 99.74% |
| Guangxi | 0.4642 | 0.0013 | 0.4630 | 0.27% | 99.73% |
| Sichuan | 0.4699 | 0.0007 | 0.4692 | 0.15% | 99.85% |
| Guizhou | 0.4517 | 0.0014 | 0.4502 | 0.32% | 99.68% |
| Shaanxi | 0.4214 | 0.0010 | 0.4203 | 0.24% | 99.76% |
| Gansu | 0.4735 | 0.0014 | 0.4721 | 0.29% | 99.71% |
| Qinghai | 0.7199 | 0.0031 | 0.7168 | 0.42% | 99.58% |
| Ningxia | 0.5009 | 0.0070 | 0.4939 | 1.39% | 98.61% |
| Fujian | 0.4251 | 0.0014 | 0.4237 | 0.32% | 99.68% |
| Shandong | 0.3249 | 0.0011 | 0.3238 | 0.35% | 99.65% |
| Yunnan | 0.4127 | 0.0008 | 0.4119 | 0.20% | 99.80% |
| Tibet | 0.4845 | 0.0022 | 0.4823 | 0.46% | 99.54% |
| Xinjiang | 0.5983 | 0.0014 | 0.5969 | 0.23% | 99.77% |
| Hubei | 0.4243 | 0.0015 | 0.4228 | 0.35% | 99.65% |
| Beijing | 0.4133 | 0.0233 | 0.3899 | 5.64% | 94.36% |
| Tianjin | 0.3601 | 0.0130 | 0.3471 | 3.61% | 96.39% |
| Hainan | 0.3241 | 0.0071 | 0.3170 | 2.20% | 97.80% |

**Table S3**. Output explanation: the emerging spatiotemporal hot spot analysis for county-level hospital beds

| Type | Practical significance | N^*^ | % |
| --- | --- | --- | --- |
| Consecutive Cold Spot | Hospital beds have been at a low level. | 120 | 20.98 |
| Consecutive Hot Spot | Hospital beds have been at a high level. | 257 | 44.93 |
| Diminishing Hot Spot | Hospital beds remain at significantly high levels for an extended period, the intensity of hotspots in the number of hospital beds per year diminishes in general, and this decrease is statistically significant. | 2 | 0.35 |
| Intensifying Hot Spot | Already 90% of the time step intervals are significant hot spots, representing at least 9 years of maintaining a high level of hospital beds and a statistically significant increase in the intensity of hospital beds hotspots. | 65 | 11.36 |
| Persistent Hot Spot | High values of hotspot intensity of hospital beds have been maintained for a long time, but the increase in hotspot intensity is not statistically significant. | 20 | 3.50 |
| Sporadic Hot Spot | Statistically significant hotspots of hospital beds occur intermittently, at irregular locations, and for years of occurrence. | 58 | 10.14 |
| New Hot Spot | For the first time in the last year, hospital beds showed high values, which have never before been clustered in hot spots. | 50 | 8.74 |
| Sum patterns |  | 572 | 24.78 |

N* indicates the number of counties in China.

**Table S4**. Relative importance of space-time explanatory factors on hospital beds from various dimensions

| Factors | VPI (median) (%) | 95% CIs (%) |
| --- | --- | --- |
| STVC model | 95.96 | 95.63-96.33 |
| Residual | 4.04 | 3.67-4.37 |
| Socioeconomic | 36.85 | 31.84-42.50 |
| Environment | 59.11 | 53.80-63.83 |
| Space | 75.71 | 68.94-81.55 |
| Time | 20.25 | 14.14-27.36 |
| X1 _space_ | 4.99 | 4.36-5.65 |
| X2 _space_ | 0.51 | 0.13-0.99 |
| X3 _space_ | 1.96 | 1.40-2.63 |
| X4 _space_ | 0.64 | 0.26-1.11 |
| X5 _space_ | 6.84 | 5.94-7.82 |
| X6 _space_ | 0.57 | 0.35-0.87 |
| X7 _space_ | 1.64 | 0.77-4.43 |
| X8 _space_ | 0.79 | 0.26-1.79 |
| X9 _space_ | 16.60 | 14.27-19.67 |
| X10 _space_ | 10.94 | 8.65-12.86 |
| X11 _space_ | 29.71 | 26.70-32.59 |
| X1 _time_ | 0.98 | 0.38-2.10 |
| X2 _time_ | 1.97 | 1.18-3.66 |
| X3 _time_ | 0.83 | 0.27-1.95 |
| X4 _time_ | 12.97 | 7.26-19.94 |
| X5 _time_ | 0.51 | 0.13-1.35 |
| X6 _time_ | 0.53 | 0.16-1.18 |
| X7 _time_ | 1.07 | 0.27-2.79 |
| X8 _time_ | 0.83 | 0.33-1.94 |
| X1 _space-time_ | 5.98 | 5.17-7.19 |
| X2 _space-time_ | 2.50 | 1.56-4.23 |
| X3 _space-time_ | 2.83 | 1.97-4.07 |
| X4 _space-time_ | 13.63 | 7.94-20.57 |
| X5 _space-time_ | 7.40 | 6.35-8.62 |
| X6 _space-time_ | 1.11 | 0.65-1.81 |
| X7 _space-time_ | 2.87 | 1.50-5.89 |
| X8 _space-time_ | 1.68 | 0.84-3.10 |
| X9 _space-time_ | 16.60 | 14.27-19.67 |
| X10 _space-time_ | 10.94 | 8.65-12.86 |
| X11 _space-time_ | 29.71 | 26.70-42.50 |


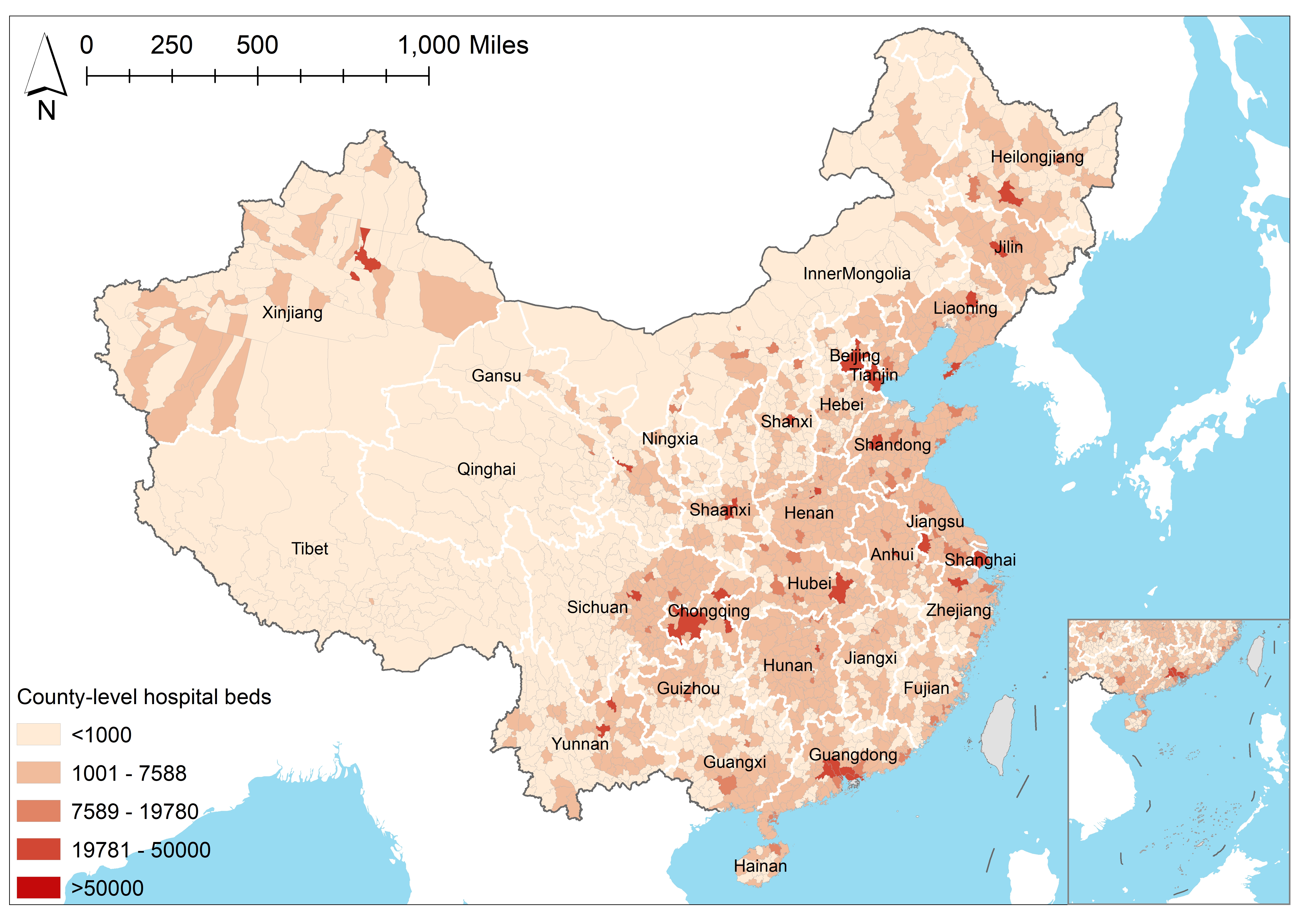


**Figure S1**. Distribution of Chinese county-level hospital beds in 2011.


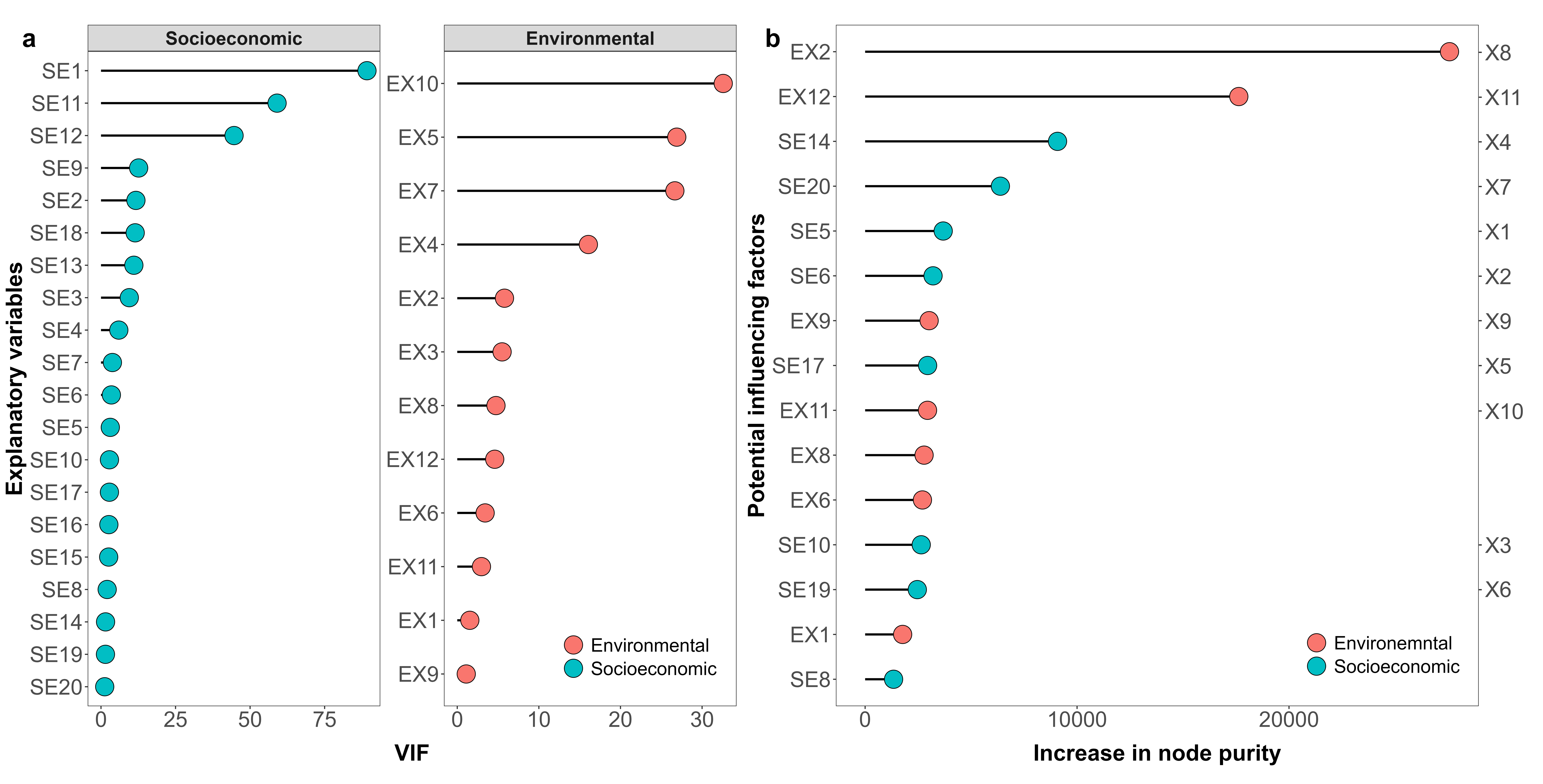


**Figure S2**. Selection of potential influencing factors: (a) the VIF of socioeconomic factors (SE1 to SE20) and environmental factors (EX1 to EX 11), and (b) the relative global contributions of candidate socioeconomic and environmental factors to hospital beds, are evaluated with the increase in node purity (a random forest-based indicator). Note that X1–X11 show the renumbering of the eleven screened factors. Influencing factors encompass: local general budget revenue per capita (X1), residents' saving deposits per capita (X2), total investment in fixed assets per capita (X3), first industry output per capita (X4), GDP per capita (X5), average urban employee wage (X6), total retail sales of consumer goods per capita (X7), nighttime light index (X8), river network density (X9), slope (X10), and road network density (X11).
